# Genetic characterization of H9N2 avian influenza viruses isolated from poultry in Poland during 2013/2014

**DOI:** 10.1007/s11262-017-1513-4

**Published:** 2017-10-19

**Authors:** Edyta Świętoń, Michał Jóźwiak, Zenon Minta, Krzysztof Śmietanka

**Affiliations:** Department of Poultry Diseases, National Veterinary Research Institute, al. Partyzantów 57, 24-100 Puławy, Poland

**Keywords:** Avian influenza, H9N2 subtype, Mutation, Phylogenetic analysis, Zoonotic potential, HA trypsin cleavage site

## Abstract

The study presents molecular characterization of H9N2 avian influenza (AI) isolates from field outbreaks in turkeys that occurred in Poland in 2013–2014. Sequences of all gene segments of one isolate from 2013 (A/turkey/Poland/14/2013(H9N2)) and two isolates from 2014 (A/turkey/Poland/08/2014(H9N2), A/turkey/Poland/09/2014(H9N2)) were obtained and analyzed in search of the phylogenetic relationship and molecular markers of zoonotic potential or increased pathogenicity. All gene segments were shown to originate from the wild bird reservoir and the close relationship of the analyzed isolates proved the link between the outbreaks in 2013 and 2014. However, remarkable molecular differences between isolates from 2013 to 2014 were identified, including mutation in the HA cleavage site (CS) leading to conversion from the PAASNR*GLF to the PAASKR*GLF motif and truncation of the PB1-F2 protein. Additionally, T97I substitution in the PA protein in A/turkey/Poland/08/2014 was detected which can be responsible for enhanced activity of viral polymerase in mammalian cells. However, experimental infection of mice with both isolates from 2014 showed their low pathogenicity, and no statistically significant differences in virus replication were observed between the viruses. Nevertheless, these findings indicate the dynamic evolution of H9N2 in the field emphasizing the need for monitoring of the situation in terms of H9N2 AI in Europe.

**Electronic supplementary material:**

The online version of this article (doi:10.1007/s11262-017-1513-4) contains supplementary material, which is available to authorized users.

## Introduction

Avian influenza virus (AIV) is an avian pathogen characterized by high diversity resulting from a high mutation rate and segmented genome enabling an exchange of genes in a reassortment event [1]. The AIV genome consists of eight negative—sense single—stranded RNA segments coding for at least 11 viral proteins [2]. The differences in antigens—haemagglutinin (HA) and neuraminidase (NA)—present on the surface of virus particles are the basis for AIV classification. Sixteen HA subtypes and nine NA subtypes have been distinguished in avian species so far [2]. Recently, the two new subtypes H17N10 and H18N11 have been detected in bats, but so far they have not been found in birds [3, 4]. The natural reservoir for all AIV subtypes is wild aquatic birds, mainly *Anseriformes* and *Charadriiformes* [5]. There have been many cases of introduction of low pathogenicity (LP) AIV of various subtypes from the wild bird reservoir to poultry, usually resulting in transient outbreaks [6]. However, selection of adaptive mutations during AIV replication in domestic birds, especially in gallinaceous poultry, may lead to the establishment of lineages circulating endemically in poultry populations. The major concern is associated with AIVs of H5 and H7 subtypes which have the capacity to become highly pathogenic upon transmission to gallinaceous poultry [1]. The most significant mutation that entails emergence of the highly pathogenic (HP) phenotype of H5 and H7 subtypes is the introduction of multiple basic amino acids at the HA cleavage site. Nonetheless, other viral genes, in particular those coding for the proteins of the polymerase complex, also carry molecular markers that influence pathogenicity, host range, and adaptation [2].

Another important AI virus is the H9N2 subtype which has been frequently detected in wild and domestic birds and has become endemic in poultry across the Middle East, Far East Asia, and North Africa since the mid-90s [1, 6, 7]. Although the H9N2 virus is of low pathogenicity and usually causes low mortality, the presence of concomitant infections may lead to high morbidity and significant economic losses. Thus, it has a considerable impact on the poultry industry [8, 9]. The hemagglutinin of influenza viruses is one of the major genetic components associated with host adaptation and even slight genetic changes can alter transmissibility [10]. Similarly, the hemagglutinins of numerous H9N2 virus strains were shown to possess human-like α2,6 SA receptor specificity resulting from the presence of leucine (L) at the position 226 of hemagglutinin and thus are subjects of concern for public health [11]. In Europe, the H9N2 subtype has been detected sporadically in wild birds and poultry; however, in recent years, a number of outbreaks in turkey flocks were reported in Germany, Italy, England, and Poland [9, 12]. In Poland, six outbreaks of H9N2 AIV were identified in two consecutive years, with four outbreaks in 2013 and two in 2014 [13]. The phylogenetic study of HA and NA sequences from the outbreaks in 2013 showed that the virus probably originated from the wild bird reservoir [9]. The objective of the present study was the analysis of all gene segments of H9N2 isolates from 2013 and 2014 to determine their relationship with other AIV strains and identify molecular markers of zoonotic potential or increased virulence. Additionally, we compared the pathogenicity of the two isolates from 2014 in a mice model, as one of the isolates possessed a mutation in the PA gene described previously as a virulence determinant for mice [14].

## Materials and methods

### RT-PCR and sequencing

The study was conducted on three isolates propagated in embryonated SPF chicken eggs: A/turkey/Poland/14/2013, A/turkey/Poland/08/2014, and A/turkey/Poland/09/2014, hereinafter referred to as A/ty/PL/14/13, A/ty/PL/08/14, and A/ty/PL/09/14, respectively. The RNA was extracted from 200 µl of infected allantoic fluid using a Viral Mini Kit (Syngen, Poland) according to the manufacturer’s protocol. The RT-PCR reactions were performed using a One Step RT-PCR Kit (Qiagen, Germany) and specific primers (sequences available upon request) which yielded partially overlapping PCR products covering the entire length of the segment. The thermal cycling conditions were as follows: reverse transcription at 50 °C for 30 min; denaturation at 95 °C for 15 min; 35 cycles of 95 °C for 45 s, 57 °C for 45 s, and 72 °C for 1 min; and a final elongation step of 72 °C for 10 min. The purified PCR products were sequenced using a BigDye Terminator v3.1 Cycle Sequencing Kit (Applied Biosystems, USA) in a 3500 Genetic Analyzer (Applied Biosystems).

### Phylogenetic and molecular analysis

Consensus sequences were assembled with LaserGene SeqMan software (version 8.1.3) (DNAStar, USA) and are available in the GenBank database under accession numbers KX470431-KX470454. The detailed information on the GenBank accession numbers is included in Table 1S in Electronic Supplementary Material (ESM). The phylogenetic and molecular analysis was performed in MEGA6 software [15] and included full coding sequences of all 8 gene segments. The phylogenetic trees for the obtained sequences and sequences from the GenBank and GISAID EpiFlu databases were constructed using the neighbor-joining method with 1000 replicates in the bootstrap test. Nucleotide sequences were translated into amino acid sequences and compared to identify the differences. The N-glycosylation sites in the HA protein were predicted using NetNGlyc software (http://www.cbs.dtu.dk/services/NetNGlyc/).

### Culture of chicken embryo fibroblasts (CEF)

CEF cells were prepared from 9 to 11-day-old SPF chicken embryos according to standard protocol. The cells were seeded into 25 cm^2^ flasks in Eagle’s minimum essential medium (MEM) containing 10% fetal bovine serum (FBS) and 1× antibiotic–antimycotic solution (Sigma-Aldrich, USA) and incubated at 37 °C in 5% CO_2_ for 24 h. Then the monolayer was washed with MEM and infected with A/ty/PL/14/13 or A/ty/PL/08/14 isolate at MOI of 1, with or without the addition of TPCK-trypsin (1 µg/ml) (Sigma-Aldrich). Stock solution of TPCK-trypsin was prepared by dissolving in MEM to a concentration of 1 mg/ml. The cultures were incubated for 1 h at 37 °C for virus adsorption; then the inoculum was replaced with maintenance medium (without FBS) with or without trypsin supplementation according to the inoculum composition. The flasks were incubated for up to 72 h. The growth of virus was confirmed by the presence of cytopathic effect (CPE) and hemagglutination (HA) assay. The culture supernatant was used for the next passage. A total of three passages were performed.

### Experimental infection of mice

Animal experiments were conducted in two groups of 6–8-week-old BALB/c mice (NVRI, Poland) infected with either A/ty/PL/08/14 or A/ty/PL/09/14. All mice were weighed before inoculation. Thirteen mice were inoculated intranasally with a dose of 10^6^ EID_50_ of virus in a volume of 50 µl. The control group consisting of five mice was inoculated with the same volume of PBS. At 1 day post infection (dpi) three naïve mice were added to each infected group to serve as control of transmission. All animals were observed for 14 days for the presence of clinical signs. At 3, 6, 10, and 14 dpi all mice were weighed and oropharyngeal swabs were collected and immersed in 1 ml of viral transport medium (Copan, Italy). Additionally, at 3, 6, and 10 dpi, three infected mice from each group were euthanized, and samples of nasal turbinates, trachea, and lungs were collected. At the end of the experiment, blood samples were collected. Before inoculation and swab collection, the mice were anesthetized with isoflurane.

Organ samples were homogenized in PBS to 20% suspension and centrifuged (10 min at 3000 x g). The RNA was extracted from the organ supernatant and swab medium as described above. A real-time RT-PCR test targeting the AIV M gene [16] was performed on all samples. The quantity of viral RNA in samples was calculated based on the standard curve generated from ten-fold dilutions of a homologous H9N2 isolate of known titer and expressed as equivalents of EID_50_ (eqEID_50_) per 0.1 g of tissue or 1 ml of swab medium. Serum samples were tested in hemagglutination inhibition (HI) assay with H9N2 antigen according to standard procedure. The differences in virus quantity between the two infected groups for each sample type collected at a given time point were tested statistically with the Mann–Whitney test and were considered statistically significant at *p* < 0.05.

## Results

### Phylogenetic analysis

**Fig. 1:**
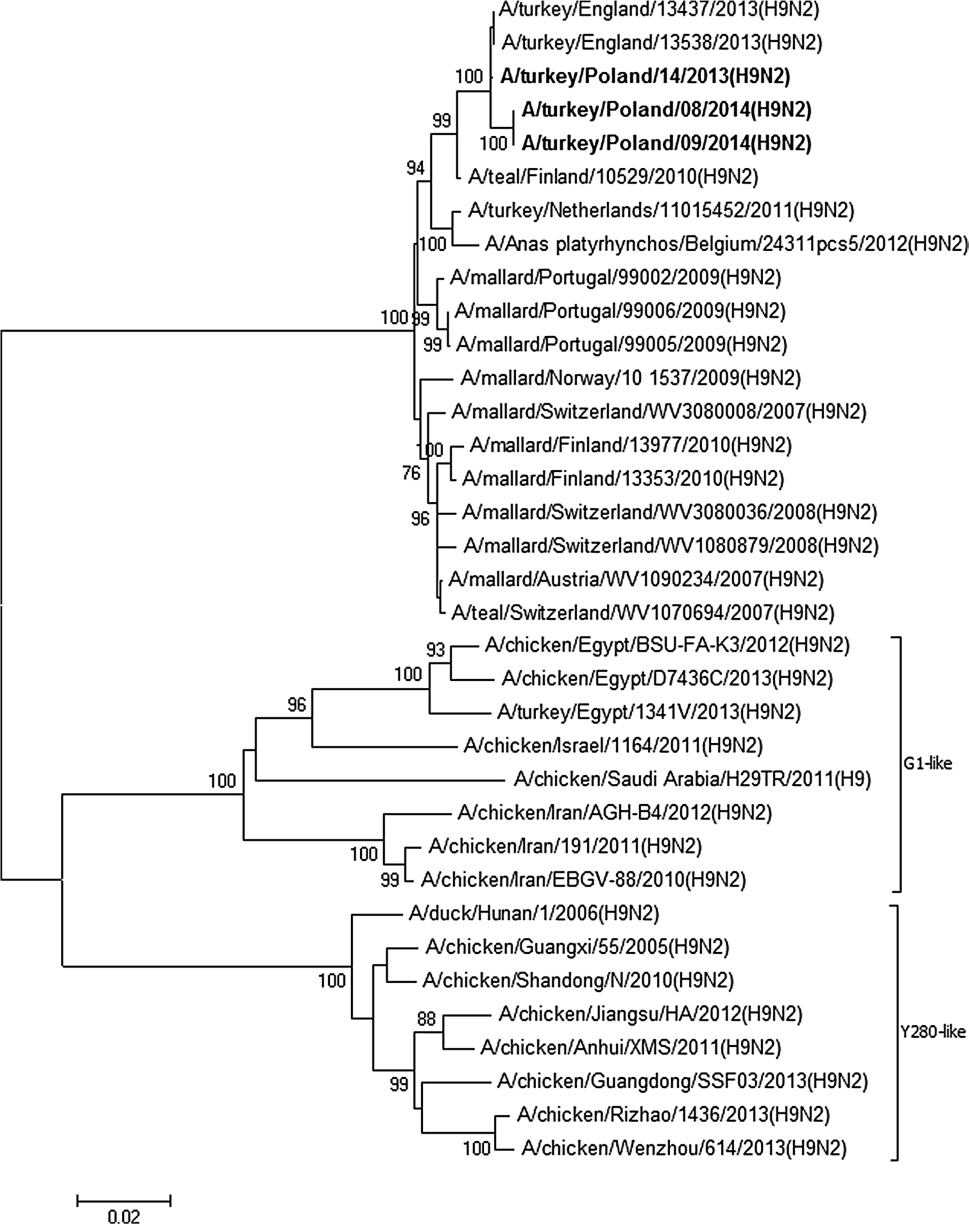
Phylogenetic tree for HA gene constructed with the neighbor-joining method. Bootstrap values ≥ 70% are shown next to the branches. The scale bar indicates the number of base substitutions per site. Analyzed strains are presented in bold

**Fig. 2:**
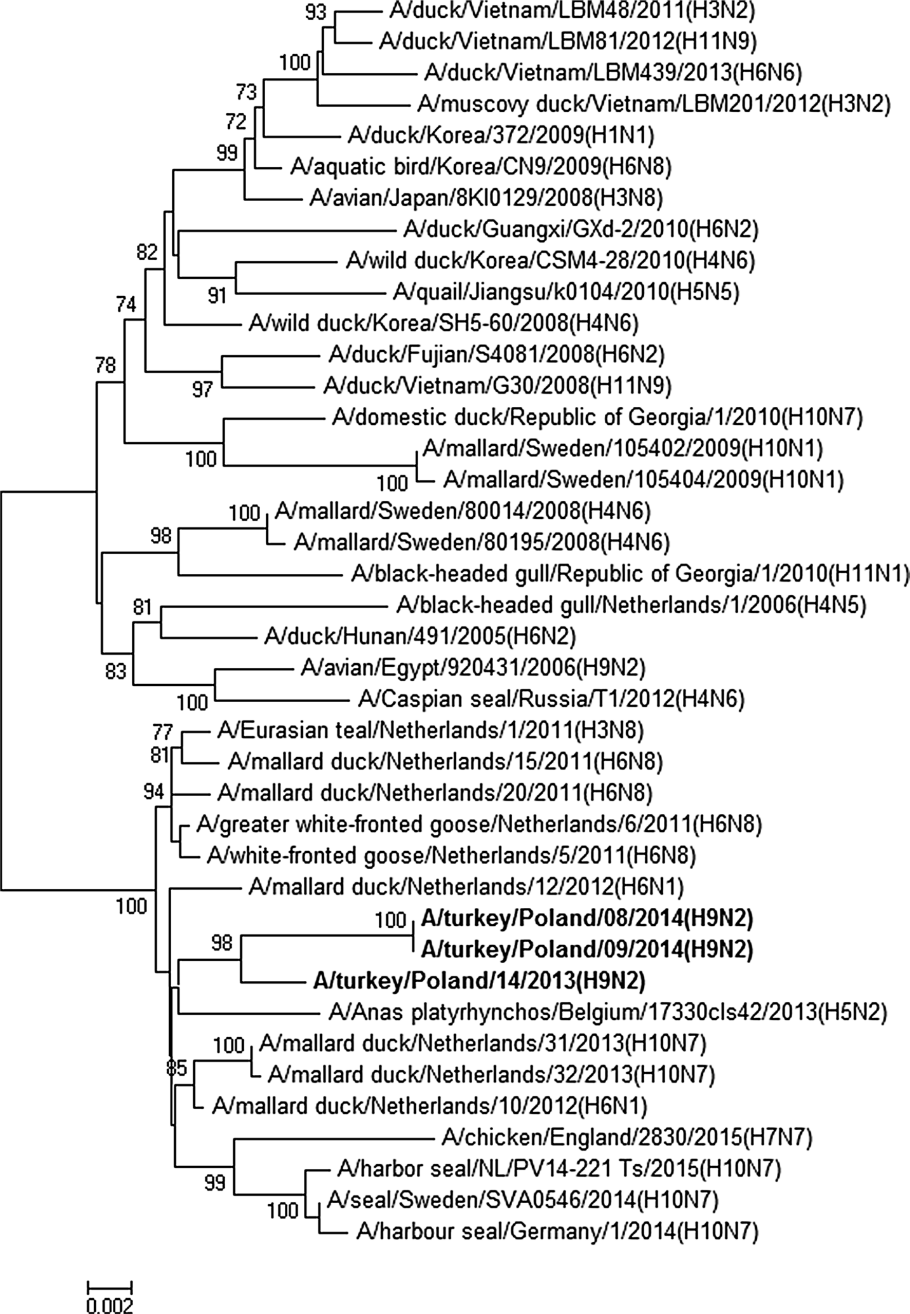
Phylogenetic tree for PB1 gene constructed with the neighbor-joining method. Bootstrap values ≥ 70% are shown next to the branches. The scale bar indicates the number of base substitutions per site. Analyzed strains are presented in bold

The nucleotide sequence identity of Polish isolates ranged from 98.9–100% for the PB1 gene to 99.7–100% for the NP gene. The location of Polish isolates on phylogenetic trees for all gene segments showed their close relationship and confirmed their wild bird origin (Figs. 1, 2, Figs. 1S–6S in ESM). In the HA and NA trees, Polish isolates clustered with H9N2 strains from an outbreak in turkeys in England and European H9N2 isolates from wild birds. The gene segments encoding internal and nonstructural proteins showed the closest relationship with mallard isolates of various subtypes detected in the Netherlands. A relationship with other poultry as well as mammalian isolates was observed for most genes. In the phylogenetic tree of the PB1 gene, the analyzed isolates grouped with an H7N7 poultry isolate and H10N7 from seals (Fig. 2). A close relationship with H10N7 from seals was also observed for the PA gene (Fig. 2S in ESM) indicating that H9N2 and H10N7 viruses share a common ancestor for both genes. In the case of PB2 gene, Polish isolates grouped with human and chicken H7N7 from Italy, whereas the NS1 gene, belonging to allele B, clustered with an H7N7 chicken isolate from England.

### Molecular analysis

Compared to isolate A/ty/PL/14/13, the isolates A/ty/PL/08/14 and A/ty/PL/09/14 showed 91 nucleotide differences each. The most variance was observed for the PB1 gene showing 25–26 nucleotide substitutions, of which 14 were synonymous, and 11–12 nonsynonymous mutations (Table 1). The mutation C170T in the PB1 gene of A/ty/PL/08/14 and A/ty/PL/09/14 caused substitution of threonine (T) with methionine (M) resulting in the simultaneous introduction of a premature stop codon at the ORF of the PB1-F2 protein and truncation of PB1-F2 to 25 amino acids (aa). On the other hand, another mutation, present also in A/ty/PL/14/13, entailed the introduction of ATG in the PB1-F2 ORF downstream of the stop codon, which can act as an additional initiation codon for the expression of the C-terminal part of PB1-F2. This mutation was synonymous in the PB1 ORF (at position 231). Other genes of the polymerase complex also showed a relatively high number of differences compared to A/ty/PL/14/13. In PA gene, 13–15 mutations were detected, of which 5–6 were nonsynonymous. PB2 showed 13–14 nucleotide differences, with 3–4 causing amino acid substitution (Table 1).

**Table 1:** Amino acid differences in analyzed H9N2 isolates

Protein	Amino acid position	A/ty/PL/14/13	A/ty/PL/08/14	A/ty/PL/09/14
PB2	376	K	R	R
	482	K	K	R/K^a^
	640	A	V	V
	717	A	V	V
PB1	20	A	T	T
	57	T	M	M
	93	A	S	S
	174	M	M/I^a^	I
	211	K	R	R
	331	E	D	D
	353	K	R	R
	528	T	A	A
	530	I	V	V
	622	C	C	C/G^a^
	634	H	Q	Q
	687	R	Q	Q
PA	4	F	L	L
	18	G	E	E
	97	T	I	T
	386	D	G	G
	407	I	V	V
	629	E	D	D
HA^b^	337	N	K	K
	445	T	I	T/I^a^
	537	L	I	I
	539	M	I	I
NP	301	I	V	V
	343	V	I	I
NA	30	A	V	V
	53	C	Y	Y
	149	L	I	I
	267	P	S	S
	329	D	N	N
NS1	60	E	G	G
NEP	39	K	R	R
M1	242	K	R	R
M2	13	N	D	D
	28	I	V	V

^a^Mixed bases at the nucleotide sequences

^b^H9 pre-HA0 numbering

Twelve nucleotide differences in the HA gene were detected, with four of them being nonsynonymous (Table 1). The analysis of amino acid sequences at the HA cleavage site showed that A/ty/PL/14/13 possessed the PAASNR*GLF motif, whereas in both isolates from 2014 asparagine (N) at position 337 (H9 pre-HA0 numbering) was substituted with lysine (K) resulting in the PAASKR*GLF motif. All analyzed isolates possessed the same amino acids at key positions at the HA receptor binding site (RBS) (R149, A150, H191, E198, L202, N232, G233, Q234, and Q235). No change in the HA glycosylation profile was observed as all isolates possessed eight putative N-glycosylation sites.

In the NA genes, five nonsynonymous and seven synonymous mutations were detected. Similarly to A/ty/PL/14/13, the isolates from 2014 also possessed a shortened NA stalk, a marker of adaptation to poultry [17]. However, the deletion encompassed 24 instead of 26 amino acids.

Fewer nucleotide differences were identified in the M, NP, and NS segments—nine, four, and four, respectively. These resulted in two amino acid changes in NP and M2 proteins and one amino acid change in NS1, NEP, and M1 proteins (Table 1).

All gene segments were also analyzed in the search for mutations associated with increased zoonotic potential. The analysis of the PA gene revealed substitution of threonine (T) with isoleucine (I) at position 97 of A/ty/PL/08/14, which has been implicated in increased virulence of AIV in mice. This implication follows from this mutation having been shown to enhance viral polymerase activity in mammalian cells, hence increasing virus replication efficiency [14, 18].

### Virus growth in the presence and absence of trypsin

Both A/ty/PL/14/13 and A/ty/PL/08/14 grew well in CEF in subsequent passages when supplemented with trypsin. The replication of both isolates was restrained in the absence of trypsin as no CPE was detected and HA tests remained negative.

### Assessment of H9N2 pathogenicity for mice

No clinical signs were apparent in any infected animals or in the control group, no weight loss was observed, and all mice survived the experiment. In the case of the A/ty/PL/08/14 group, viral RNA was detected in nasal turbinate at 3 and 6 dpi, and in trachea, lungs, and swabs at 3, 6, and 10 dpi (Table 2). In the A/ty/PL/09/14 group, viral RNA was detected in nasal turbinate and trachea samples at 3 and 6 dpi, and in lungs and swabs at 3, 6, and 10 dpi (Table 2). No virus shedding was detected at 14 dpi. The statistical analysis did not demonstrate the differences between both groups to be statistically significant as p-values exceeded 0.05. The HI titers ranged from 4 to 16 in the A/ty/PL/08/14 group and < 4 to 8 in the A/ty/PL/09/14 group. No virus shedding, viral RNA in organ samples, or seroconversion were detected in contact mice.

**Table 2:** The quantity of viral RNA in oropharyngeal swabs (OS), nasal turbinates (NT), trachea (T), and lungs (L) collected at 3, 6, and 10 dpi from mice infected with H9N2 isolates

Isolate	Sample	3 dpi		6 dpi		10 dpi	
		pos/tested^1^	Mean (± SD)^b^	pos/tested^a^	Mean (± SD)^b^	pos/tested^a^	Mean (± SD)^b^
A/ty/PL/08/14	OS	8/13	1.5 × 10^1^ (± 2.0 × 10^1^)	1/10	3.1 (± 9.8)	1/7	4.36 (± 11.5)
	NT	3/3	1.1 × 10^2^ (± 1.4 × 10^2^)	3/3	2.0 × 10^1^ (± 5.1)	0/3	–
	T	3/3	8.3 × 10^4^ (± 1.2 × 10^5^)	3/3	1.6 × 10^3^ (± 1.7 × 10^3^)	1/3	4.5 (± 7.8)
	L	3/3	1.7 × 10^4^ (± 1.3 × 10^4^)	3/3	2.5 × 10^4^ (± 2.4 × 10^4^)	2/3	60.2 (± 83.9)
A/ty/PL/09/14	OS	6/13	2.1 × 10^1^ (± 3.7 × 10^1^)	1/10	0.82 (± 2.6)	1/7	5.29 (± 13.98)
	NT	2/3	5.1 (± 4.6)	2/3	6.32 (± 5.56)	0/3	–
	T	3/3	1.7 × 10^4^ (± 2.8 × 10^4^)	3/3	2.1 × 10^2^ (± 3.3 × 10^2^)	0/3	–
	L	3/3	7.7 × 10^3^ (± 9.0 × 10^3^)	3/3	7.7 × 10^3^ (± 1.3 × 10^4^)	1/3	17.88 (± 30.97)

^a^Number of positive samples/number of tested samples

^b^eqEID_50_ per 0.1 g of tissue or 1 ml of swab medium; SD—standard deviation

## Discussion

The H9N2 subtype is one of the most common AIV occurring in poultry in the world. Although it wreaks less harm than H5N1, or than clade 2.3.4.4 H5Nx recently has, it can have a significant impact on poultry production as an exacerbating factor or contribute to the emergence of novel AIV lineages with human health implications [7, 19]. Although H9N2 AIV in Europe has not been shown to be associated with endemic lineages circulating in Asia and Africa [9, 12], recurrent outbreaks have raised questions about the virus’ evolution and its capacity for crossing the host species barrier.

The phylogenetic analysis of HA and NA genes from the 2013 outbreaks in Poland [9] was complemented by an analysis of the gene segments encoding internal and nonstructural proteins, and also included isolates from 2014 to investigate their relationship, evolution, and presence of molecular markers of zoonotic potential. The close relationship of the Polish H9N2 isolates observed for each gene segment indicated that all H9N2 outbreaks in Poland are a part of one epidemic that probably began in 2012. As evidenced by the HA and NA trees, the same lineage caused an outbreak in turkeys in England in 2013 [12]. Furthermore, the remaining gene segments were shown to originate from LPAI strains circulating in recent years in wild birds which corroborated the findings for the HA and NA genes presented in the previous study [9].

Despite close relatedness, significant molecular differences between the isolates from 2013 and 2014 were identified, indicating dynamic virus evolution. The isolates from 2014 showed a truncated PB1-F2 protein which terminated after 25 aa. Alteration in the PB1-F2 length is rarely observed in avian influenza isolates, as the overwhelming majority (~ 90%) possesses the full-length protein of 90 amino acids. In contrast, in 96% of human and 74% of swine H1N1 isolates PB1-F2 mostly terminates after 57 and 52 aa, respectively [20]. Previous studies indicated that the diversity of PB1-F2 results from purifying selection on PB1, since synonymous substitutions of the third codon position in the PB1 ORF cause nonsynonymous mutations in the second base in the PB1-F2 ORF [21]. However, in this case, the nonsense mutation in PB1-F2 corresponds to a nonsynonymous substitution in the PB1, which is rather unusual [21]. On the other hand, another initiation codon in the PB1-F2 ORF may result in the expression of an N-terminally truncated protein compensating for the premature stop codon [22]. This signature was already present in A/ty/PL/14/13 and only a few isolates from the GenBank database possess this additional ATG in the PB1-F2 ORF (data not shown). The role of PB1-F2 in influenza virus pathogenicity seems to depend strongly on the virus strain and the host [23]. In the case of avian species, knowledge of the biological function of PB1-F2 is scant. However, in some studies on gallinaceous poultry, the PB1-F2-deficient AIVs showed increased pathogenicity which suggests that PB1-F2 may be an alleviating factor contributing to host survival and hence more effective transmission [23–25]. More investigations are necessary to determine the role of PB1-F2 truncation in the analyzed isolates and the possibility of the expression of C-terminal PB1-F2 from the downstream ATG. A possible means of carrying out that investigation would be constructing a recombinant virus based on the isolate from 2014 coding full PB1-F2 and a variant with completely knocked-out PB1-F2 expression. Comparison of their virulence for turkeys and assessment of PB1-F2 expression by western blotting would contribute to the knowledge of the significance of PB1-F2 for AIV.

Since the HA cleavage site plays a key role in the pathogenicity of AIV of H5 and H7 subtypes, the sequence of amino acids at this HA domain is the primary molecular marker examined for pathogenicity assessment. The HA cleavage sites in subtypes other than H5 or H7 are well conserved as LPAI viruses usually have a monobasic cleavage site [2]. However, the majority of endemic G1 and Y280 H9N2 viruses from Asia and Africa have a dibasic (RSSR*GLF) or tribasic (RSK/RR*GLF) motif, which have been shown to be susceptible to cleavage by trypsin-like proteases such as matriptase or by furin, thus increasing the spectrum of enzymes able to activate the HA protein [26, 27]. This trait is believed to be a result of adaptation of H9N2 viruses to land-based poultry [28]. The emergence of an additional basic amino acid at the HA cleavage site of the Polish isolates does not seem to influence the pathogenicity since the intravenous pathogenicity index (IVPI) tests for all analyzed strains resulted in a value of 0.0 (data not published) and the virus is incapable of replication in the absence of trypsin. However, this mutation may determine a gain in virus fitness and further experiments with recombinant viruses differing only with position 337 in the HA protein, both in an animal model to compare their replication efficiency, and assessment of HA cleavability in vitro, will elucidate its significance.

Another key region of the HA protein is the RBS responsible for recognition and attachment to cell receptors. Amino acids in particular positions of HA RBS determine the affinity to human- or avian-like sialic acid receptors. Position 226 according to H3 numbering (234 in H9 numbering) is the most significant as it was shown that the presence of L226 results in better affinity for human-type receptors, whereas Q226 determines avian-like receptor specificity [11]. HA-L226 in most viruses of G1 and Y280 lineage is treated as the main molecular marker of their zoonotic potential, but a recent study showed no apparent association of position 226 with human-like receptor affinity, position 190 being involved instead [29]. Similarly, a study by Teng et al. showed that valine (V) at position 190 of HA is involved in increased binding to human and mouse cells, without altering receptor binding specificity [30]. The HA-190V has been fixed in H9N2 viruses over the last years, and more importantly, most human H9N2 isolates possess this mutation [30]. Human infections with AIV H9N2 have been reported occasionally, with usually mild influenza-like symptoms [31]. However, studies on the prevalence of antibodies to the H9N2 virus in human populations with occupational poultry exposure showed that infections occur more often but are probably asymptomatic or clinically indistinguishable from seasonal influenza or the common cold, and therefore remain unrecognized [32, 33]. The RBS of analyzed isolates maintained an avian-like profile and was identical to patterns previously observed in other European H9 AIVs related to Y439-like lineage [28], and V190 in the HA was not observed. The HA glycosylation pattern was also specific for strains belonging to the Y439-like lineage [28]. Nevertheless, monitoring the sialic acid receptor preference is of great significance since it provides information on the emergence of potentially zoonotic strains.

The NA gene of Polish isolates showed a deletion in the stalk region which is a marker of adaptation to poultry [17], but some difference in the length of this deletion was observed. This may suggest that the isolates from 2013 and 2014 are derived from strains that took separate evolution tracks after splitting from a common ancestor bearing the full-length NA gene.

The human health concern associated with Asian H9N2 viruses results not only from its human-type receptor specificity but also from the detection of their segments coding internal and nonstructural proteins in human H7N9 and H10N8 viruses [19, 34]. H9N2 AIVs were identified in other mammalian hosts such as mink and pigs [35, 36]. Serological investigations proved that H9N2 AIV infections occur also in foxes and raccoon dogs, and are effectively transmitted from mink to mink, fox, and raccoon dog [36]. Furthermore, an increase in prevalence of mutations in the polymerase complex responsible for enhanced viral polymerase activity in mammalian cells has been observed in recent years in H9N2 AIV, and they are also common in human H7N9 and H10N8 viruses [37, 38].

The search for markers of increased zoonotic potential showed the presence of T97I substitution in the PA gene of A/ty/PL/08/14. The emergence of this mutation was observed during adaptation of AIV isolates of various subtypes (H5N2, H6N1, H10N7, and H7N9) to mice [14, 18, 39, 40] suggesting that PA-97I is a general determinant of pathogenicity in mice. Variants possessing PA-97I were also recovered after experimental infection of mice with HPAIV H5N1 and H7N1 [41, 42]. Natural occurrence of this mutation without prior adaptation was reported in a field strain of H6N5 AIV which caused severe disease and mortality in mice and was also capable of efficient replication and transmission in ferrets [43]. There is no evidence for mammalian adaptation of A/ty/PL/08/14, and no other molecular traits associated with increased pathogenicity for mammals have been identified. Nevertheless, this prompted us to investigate whether the presence of this mutation confers any gain in virulence for mice. It was achieved by comparison of the clinical outcome of infection in mice with A/ty/PL/08/14 and A/ty/PL/09/14, since the two isolates differed in almost nothing but the PA-T97I mutation. Neither isolate showed high pathogenicity for mice since no clinical signs or mortality were observed, and transmission between infected and contact mice did not occur. Viral RNA in lungs was detected until 10 dpi in both groups, whereas in the trachea and nasal turbinates virus clearance was faster. Although differences in the quantity of viral RNA between A/ty/PL/08/14 and A/ty/PL/09/14 were noticeable, they were not statistically significant. This suggests that the presence of PA-97I in A/ty/PL/08/14 is insufficient for better adaptation to mice, but because of dynamic evolution observed in the field, the emergence of additional determinants of zoonotic potential cannot be excluded and should be further monitored.

## Electronic supplementary material

Below is the link to the electronic supplementary material.

Supplementary material 1 (DOCX 53 kb)

## References

[1] Swayne DE, Suarez DL, Swayne DE (2013). L.D. Sims. Diseases of Poultry.

[2] Perdue ML, Swayne DE (2008). Avian Influenza.

[3] Tong S, Li Y, Rivailler P, Conrardy C, Castillo DA, Chen LM, Recuenco S, Ellison JA, Davis CT, York IA, Turmelle AS, Moran D, Rogers S, Shi M, Tao Y, Weil MR, Tang K, Rowe LA, Sammons S, Xu X, Frace M, Lindblade KA, Cox NJ, Anderson LJ, Rupprecht CE, Donis RO (2012). Proc. Natl. Acad. Sci. U.S.A..

[4] Tong S, Zhu X, Li Y, Shi M, Zhang J, Bourgeois M, Yang H, Chen X, Recuenco S, Gomez J, Chen LM, Johnson A, Tao Y, Dreyfus C, Yu W, McBride R, Carney PJ, Gilbert AT, Chang J, Guo Z, Davis CT, Paulson JC, Stevens J, Rupprecht CE, Holmes EC, Wilson IA, Donis RO (2013). PLoS Pathog..

[5] Munster VJ, Baas C, Lexmond P, Waldenstrom J, Wallensten A, Fransson T, Rimmelzwaan GF, Beyer WE, Schutten M, Olsen B, Osterhaus AD, Fouchier RA (2007). PLoS Pathog..

[6] Brown IH (2010). Avian Dis..

[7] Monne I, Hussein HA, Fusaro A, Valastro V, Hamoud MM, Khalefa RA, Dardir SN, Radwan MI, Capua I, Cattoli G (2013). Influenza Other Respir. Viruses.

[8] Pan Q, Liu A, Zhang F, Ling Y, Ou C, Hou N, He C (2012). BMC Vet. Res..

[9] Śmietanka K, Minta Z, Świętoń E, Olszewska M, Jóźwiak M, Domańska-Blicharz K, Wyrostek K, Tomczyk G, Pikuła A (2014). Avian Pathol..

[10] Tumpey TM, Maines TR, Van Hoeven N, Glaser L, Solórzano A, Pappas C, Cox NJ, Swayne DE, Palese P, Katz JM, García-Sastre A (2007). Science.

[11] Wan H, Perez DR (2007). J. Virol..

[12] Reid SM, Banks J, Ceeraz V, Seekings A, Howard WA, Puranik A, Collins S, Manvell R, Irvine RM, Brown IH (2016). Avian Dis..

[13] Śmietanka K, Minta Z (2014). Acta Biochim. Pol..

[14] Song MS, Pascua PN, Lee JH, Baek YH, Lee OJ, Kim CJ, Kim H, Webby RJ, Webster RG, Choi YK (2009). J. Virol..

[15] Tamura K, Stecher G, Peterson D, Filipski A, Kumar S (2013). Mol. Biol. Evol..

[16] Spackman E, Senne DA, Myers TJ, Bulaga LL, Garber LP, Perdue ML, Lohman K, Daum LT, Suarez DL (2002). J. Clin. Microbiol..

[17] Munier S, Larcher T, Cormier-Aline F, Soubieux D, Su B, Guigand L, Labrosse B, Cherel Y, Quéré P, Marc D, Naffakh N (2010). J. Virol..

[18] Cheng K, Yu Z, Chai H, Sun W, Xin Y, Zhang Q, Huang J, Zhang K, Li X, Yang S, Wang T, Zheng X, Wang H, Qin C, Qian J, Chen H, Hua Y, Gao Y, Xia X (2014). Virology.

[19] Yu X, Jin T, Cui Y, Pu X, Li J, Xu J, Liu G, Jia H, Liu D, Song S, Yu Y, Xie L, Huang R, Ding H, Kou Y, Zhou Y, Wang Y, Xu X, Yin Y, Wang J, Guo C, Yang X, Hu L, Wu X, Wang H, Liu J, Zhao G, Zhou J, Pan J, Gao GF, Yang R, Wang J (2014). J. Virol..

[20] Dundon WG (2012). Virus Genes.

[21] Zell R, Krumbholz A, Eitner A, Krieg R, Halbhuber KJ, Wutzler P (2007). J. Gen. Virol..

[22] Zamarin D, Ortigoza MB, Palese P (2006). J. Virol..

[23] Deventhiran J, Kumar SR, Raghunath S, Leroith T, Elankumaran S (2015). J. Virol..

[24] James J, Howard W, Iqbal M, Nair VK, Barclay WS, Shelton H (2016). J. Gen. Virol..

[25] Leymarie O, Embury-Hyatt C, Chevalier C, Jouneau L, Moroldo M, Da Costa B, Berhane Y, Delmas B, Weingartl HM, Le Goffic R (2014). PLoS ONE.

[26] Tse LV, Hamilton AM, Friling T, Whittaker GR (2014). J. Virol..

[27] Baron J, Tarnow C, Mayoli-Nüssle D, Schilling E, Meyer D, Hammami M, Schwalm F, Steinmetzer T, Guan Y, Garten W, Klenk HD, Böttcher-Friebertshäuser E (2013). J. Virol..

[28] Slomka MJ, Hanna A, Mahmood S, Govil J, Krill D, Manvell RJ, Shell W, Arnold ME, Banks J, Brown IH (2013). Vet. Microbiol..

[29] Peacock TP, Benton DJ, Sadeyen JR, Chang P, Sealy JE, Bryant JE, Martin SR, Shelton H, McCauley JW, Barclay WS, Iqbal M (2017). Emerg Microbes Infect..

[30] Teng Q, Xu D, Shen W, Liu Q, Rong G, Li X, Yan L, Yang J, Chen H, Yu H, Ma W, Li Z (2016). J. Virol..

[31] Sun Y, Liu J (2015). Protein Cell.

[32] Li S, Zhou Y, Song W, Pang Q, Miao Z (2016). J. Med. Virol..

[33] de Bruin E, Zhang X, Ke C, Sikkema R, Koopmans M (2017). Zoonoses Public Health.

[34] Ye G, Liang CH, Hua DG, Song LY, Xiang YG, Guang C, Lan CH, Ping HY (2016). Front Microbiol..

[35] Yu H, Zhou YJ, Li GX, Ma JH, Yan LP, Wang B, Yang FR, Huang M, Tong GZ (2011). Vet. Microbiol..

[36] Yong-Feng Z, Fei-Fei D, Jia-Yu Y, Feng-Xia Z, Chang-Qing J, Jian-Li W, Shou-Yu G, Kai C, Chuan-Yi L, Xue-Hua W, Jiang SJ, Zhi-Jing X (2017). Sci. Rep..

[37] Xu G, Zhang X, Gao W, Wang C, Wang J, Sun H, Sun Y, Guo L, Zhang R, Chang KC, Liu J, Pu J (2016). J. Virol..

[38] Xiao C, Ma W, Sun N, Huang L, Li Y, Zeng Z, Wen Y, Zhang Z, Li H, Li Q, Yu Y, Zheng Y, Liu S, Hu P, Zhang X, Ning Z, Qi W, Liao M (2016). Sci Rep.

[39] Wu H, Peng X, Peng X, Cheng L, Jin C, Lu X, Xie T, Yao H, Wu N (2016). Arch. Virol..

[40] Zhao Y, Yu Z, Liu L, Wang T, Sun W, Wang C, Xia Z, Gao Y, Zhou B, Qian J, Xia X (2016). Vet. Microbiol..

[41] Lipatov AS, Krauss S, Guan Y, Peiris M, Rehg JE, Perez DR, Webster RG (2003). J. Virol..

[42] Rigoni M, Shinya K, Toffan A, Milani A, Bettini F, Kawaoka Y, Cattoli G, Capua I (2007). Virology.

[43] Nam JH, Kim EH, Song D, Choi YK, Kim JK, Poo H (2011). J. Virol..

