# Supplementary material for: Genetic characterization of H9N2 avian influenza viruses isolated from poultry in Poland during 2013/2014
Source: Virus Genes. 2017 Oct 19;54(1):67–76. doi: 10.1007/s11262-017-1513-4 (PMC5847159; doi:10.1007/s11262-017-1513-4)
Supplement: Supplementary file 1 — Supplementary material 1 (DOCX 53 kb) [file 11262_2017_1513_MOESM1_ESM.docx]

Electronic supplementary material

**Genetic characteristics of H9N2 avian influenza isolates from poultry outbreaks in Poland**

Edyta Świętoń*, Michał Jóźwiak, Zenon Minta, Krzysztof Śmietanka

Department of Poultry Diseases, National Veterinary Research Institute, al. Partyzantów 57, 24-100 Puławy, Poland,

*corresponding author, e-mail: edyta.swieton@piwet.pulawy.pl

Table 1S. GenBank accession numbers for gene segments of H9N2 isolates from Poland.

| Isolate  Segment | A/ty/PL/14/13 | A/ty/PL/08/14 | A/ty/PL/09/14 |
| --- | --- | --- | --- |
| PB2 | KX470431 | KX470439 | KX470447 |
| PB1 | KX470432 | KX470440 | KX470448 |
| PA | KX470433 | KX470441 | KX470449 |
| HA | KX470434 | KX470442 | KX470450 |
| NP | KX470435 | KX470443 | KX470451 |
| NA | KX470436 | KX470444 | KX470452 |
| M | KX470437 | KX470445 | KX470453 |
| NS | KX470438 | KX470446 | KX470454 |

Fig. 1S. Phylogenetic tree for NA gene constructed with the neighbor-joining method. Bootstrap values of ≥70% are shown next to the branches. The scale bar indicates number of base substitutions per site. Analyzed strains are presented in bold.

Fig. 2S. Phylogenetic tree for PA gene constructed with the neighbor-joining method. Bootstrap values of ≥70% are shown next to the branches. The scale bar indicates number of base substitutions per site. Analyzed strains are presented in bold.

Fig. 3S. Phylogenetic tree for PB2 gene constructed with the neighbor-joining method. Bootstrap values of ≥70% are shown next to the branches. The scale bar indicates number of base substitutions per site. Analyzed strains are presented in bold.

Fig. 4S. Phylogenetic tree for NP gene constructed with the neighbor-joining method. Bootstrap values of ≥70% are shown next to the branches. The scale bar indicates number of base substitutions per site. Analyzed strains are presented in bold.

Fig. 5S. Phylogenetic tree for NS gene constructed with the neighbor-joining method. Bootstrap values of ≥70% are shown next to the branches. The scale bar indicates number of base substitutions per site. Analyzed strains are presented in bold.

Fig. 6S. Phylogenetic tree for M gene constructed with the neighbor-joining method. Bootstrap values of ≥70% are shown next to the branches. The scale bar indicates number of base substitutions per site. Analyzed strains are presented in bold.
